# Evaluation of UBE3A antibodies in mice and human cerebral organoids

**DOI:** 10.1038/s41598-021-85923-x

**Published:** 2021-03-18

**Authors:** Dilara Sen, Zuzana Drobna, Albert J. Keung

**Affiliations:** 1 Department of Chemical and Biomolecular Engineering, North Carolina State University, Raleigh, NC 27695-7905 USA; 2 Department of Biological Sciences, North Carolina State University, Raleigh, NC 27695-7614 USA

**Keywords:** Pluripotent stem cells, Biological models, Experimental organisms

## Abstract

UBE3A is an E3 ubiquitin ligase encoded by the neurally imprinted *UBE3A* gene. The abundance and subcellular distribution of UBE3A has been the topic of many previous studies as its dosage and localization has been linked to neurodevelopmental disorders including Autism, Dup15q syndrome, and Angelman syndrome. While commercially available antibodies have been widely employed to determine UBE3A localization, an extensive analysis and comparison of the performance of different UBE3A antibodies has not been conducted. Here we evaluated the specificities of seven commercial UBE3A antibodies in two of the major experimental models used in UBE3A research, mouse and human pluripotent stem cell-derived neural cells and tissues. We tested these antibodies in their two most common assays, immunofluorescence and western blot. In addition, we also assessed the ability of these antibodies to capture dynamic spatiotemporal changes of UBE3A by utilizing human cerebral organoid models. Our results reveal that among the seven antibodies tested, three antibodies demonstrated substantial nonspecific immunoreactivity. While four antibodies show specific localization patterns in both mouse brain sections and human cerebral organoids, these antibodies varied significantly in background signals and staining patterns in undifferentiated human pluripotent stem cells.

## Introduction

UBE3A is a HECT domain E3 ubiquitin ligase that targets substrate proteins, including itself, for proteasomal degradation^1^. In addition to this role, UBE3A also has a putative role in transcriptional regulation where it co-activates steroid hormone receptors such as progesterone and estrogen^2^. Although biallelic in most tissues, UBE3A is expressed exclusively from the maternal allele in neurons due to tissue specific paternal imprinting^3^. Mutations or deletions of maternal *UBE3A*, leads to loss of UBE3A in neurons and Angelman syndrome (AS), a severe neurodevelopmental disorder characterized by delayed development, speech impairment, ataxia, and intellectual disability^4^. On the other hand, increased levels of UBE3A activity due to gene duplication or mutation has been associated with Dup15q syndrome and Autism Spectrum Disorder (ASD)^5–7^.

In addition to its imprinted expression, one of the key molecular features of UBE3A is its distinct subcellular localization patterns, potentially regulated by its three isoforms^8,9^. Although it has a cytoplasmic component, UBE3A is predominantly nuclear in human and mouse neurons^10–13^; however, its roles in these subcellular compartments in different cell types and developmental stages have not been fully uncovered. Previous reports show that while mice lacking the nuclear UBE3A isoform (mIso3) exhibit electrophysiological and behavioral deficits similar to other AS model mice^13^, the cytoplasmic UBE3A isoform (mIso2) is important for polarized dendrite morphogenesis^14^. Taken together, these findings indicate that both cytoplasmic and nuclear components of UBE3A may be critical for proper brain development and underscore the importance of studying its subcellular distribution and abundance.

The primary methods to determine UBE3A localization have been the use of commercially available antibodies in immunoassays such as western blot, subcellular fractionation followed by immunoblotting, or immunostaining of fixed cell culture or tissue samples. Although a few commercial UBE3A antibodies have been rigorously validated in the published literature for mouse brain tissue^12^ and human stem cell derived neurons^9^, others are less well documented. Furthermore, except for the mouse monoclonal antibody from Sigma-Aldrich (SAB1404508)^9^ and rabbit polyclonal antibody from Bethyl Laboratories (A300-351A)^15^, the specificities of most antibodies in human samples remain unknown, and even when validated, were tested only in limited cell types. With the growing use of human pluripotent stem cell-derived neuronal cultures and three-dimensional cerebral organoids as models to study human UBE3A biology, it will be important to assess the specificities of UBE3A antibodies in human stem cell-derived systems as well as mouse models. Human stem cells and cerebral organoids are therefore the major focus of this work, with the use of mouse tissues to provide comparisons to prior work and to identify potential species-specific differences.

In this work we evaluated seven commercial UBE3A antibodies including those most commonly used in the field. We tested antibody specificities in immunofluorescence (IF) and western blotting (WB) assays using AS model mice and the H9_*UBE3A* m−/p−_ hESC line as negative controls. In addition to specificity, we also evaluated the ability of these antibodies to capture important disease relevant features of UBE3A through the use of whole brain human cerebral organoid (hCO) models generated from a large panel of human pluripotent stem cell (hPSC) lines including isogenic wild-type (H9_WT_), AS (H9_*UBE3A* m−/p+_), and double knockout models (H9_*UBE3A* m−/p−_), two additional WT hPSC lines (H1_WT_ and an hiPSC line derived from a neurotypical donor, hiPSC_WT_), and two patient-derived AS hiPSC lines (derived from a patient with a large chromosome15: q11-13 deletion, hiPSC_ASdel_, and F583S point mutation, hiPSC_ASmut_).

## Results

### Four commercial UBE3A antibodies show specific and similar localization patterns in mouse brain sections

**Figure 1:**
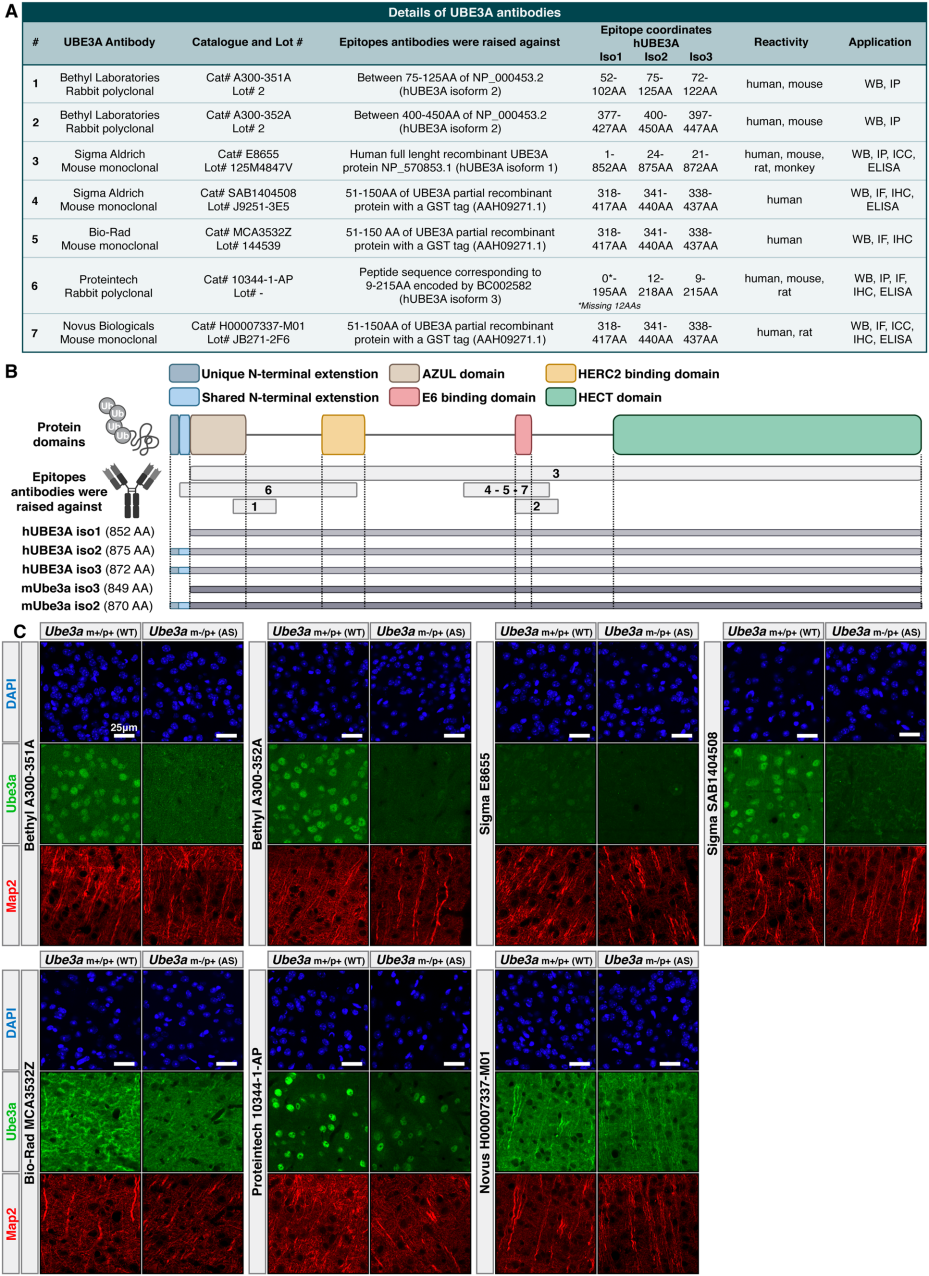
Four commercial UBE3A antibodies show specific and similar localization patterns in mouse brain sections. (**A**) Details of commercial UBE3A antibodies used in this study. (**B**) Schematic representation of the UBE3A protein, its known functional domains, epitopes used to raise the antibodies used in this study, and corresponding regions in different protein isoforms. Depicted are the N-terminal extensions of UBE3A mouse isoform 2 (mUBE3A Iso2) and human isoform 2 and 3 (hUBE3A Iso3), the N-terminal zinc-binding AZUL domain, HERC2 binding domain, E6 binding domain and the C-terminal HECT domain. (**C**) Immunofluorescence analysis from wild-type *(Ube3a m*+*/p*+*)* and Angelman Syndrome *(Ube3a m−/p*+*)* mouse models using seven different UBE3A antibodies.

In this study we evaluated seven different commercially available UBE3A antibodies from five vendors (Fig. 1A,B and S1A). The criteria of selection for the antibodies ranged from their previous use in the literature, applications reported as appropriate by the vendors, and the epitopes they were raised against. The epitopes these antibodies were raised against cluster around two functional UBE3A domains: the AZUL domain that was recently implicated in protein localization^13^ and the E6 oncoprotein binding domain (Figs. 1B and S1A). While six of the selected antibodies target common regions in all protein coding UBE3A isoforms (human isoforms hUBE3A iso1,2,3 and mouse isoforms mUbe3a iso2,3), the Proteintech UBE3A antibody was raised against an epitope which includes the 12 amino acid region that is unique to the long UBE3A isoforms (hUBE3A iso2-3 and mUbe3a iso2) (Figs. 1A,B and S1A).

We first evaluated the specificity of the selected antibodies in an IF assay with adult (P191) WT (*Ube3a*_m+/p+_) and AS (*Ube3a*_m−/p+_) mouse brain sections. Mouse models are widely used in the field and they have been shown to successfully model some of the core molecular features of UBE3A including imprinting of the paternal allele and nuclear localization of Ube3a in neurons^16^. They have also been extensively studied using IF, especially with the Sigma-Aldrich antibody (SAB1404508), and would therefore serve as a point of comparison for our IF methodology which closely mirrors prior work^12^. We focused specifically on cortical regions with a high density of neurons, as indicated by MAP2 staining, that previous work showed exhibits strong nuclear Ube3a signal in WT mice and no Ube3a signal in AS mice^10,12^ (Figs. 1C and S1B).

Our analysis showed that the antibodies from Bethyl Laboratories performed similarly to the commonly used Sigma-Aldrich antibody (SAB1404508) and exhibited the expected prominent nuclear localization pattern in WT neurons (Fig. 1C). Moreover, all three antibodies showed drastically reduced immunoreactivity in AS model mice (*Ube3a*_m−/p+_) due to paternal imprinting. Among them, the Sigma-Aldrich antibody (SAB1404508) demonstrated the lowest background signal followed by Bethyl Laboratories A300-352A and Bethyl Laboratories A300-351A (Fig. 1C). In addition to these three antibodies, the other Sigma-Aldrich antibody (E8655) also showed specific, but weaker staining in neuronal nuclei (Fig. 1C). While the Bio-Rad and Novus Biologicals antibodies did not show any specific staining pattern, the Proteintech antibody showed the expected nuclear labeling in WT mouse neurons. Surprisingly, AS sections also showed a very similar nuclear staining pattern with the Proteintech antibody indicating its potential nonspecific labeling (Fig. 1C).

### Commercial antibodies vary significantly in background signal in double *UBE3A* knockout pluripotent stem cells

**Figure 2:**
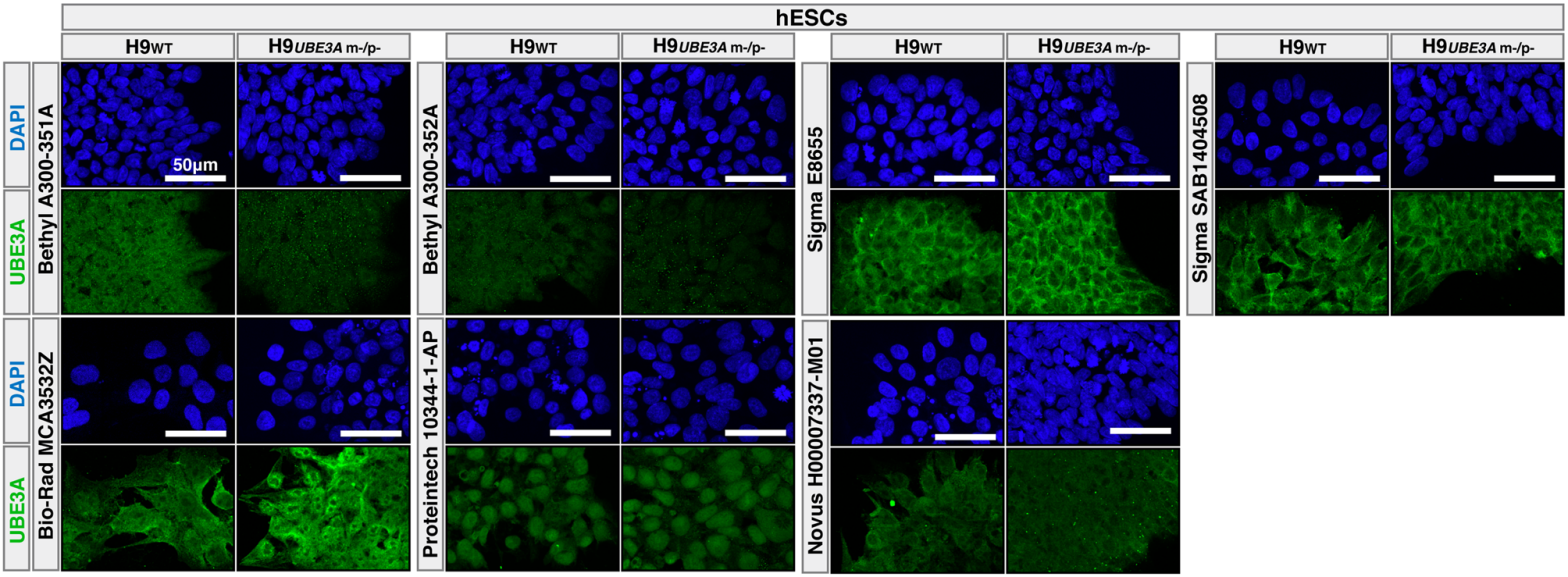
Commercial antibodies vary significantly in background signal in double *UBE3A* knockout pluripotent cells. Immunofluorescence analysis of H9 wild type (H9_WT_) and H9 double *UBE3A* knockout (H9_*UBE3 m−/p−*_) hESCs using seven different UBE3A antibodies.

Mouse models were central to many pivotal discoveries in the field. However, given the 100 million years of divergent evolution between rodents and primates, the patterns of UBE3A expression in humans may not be fully conserved. Recent studies support this notion where differences in the localization patterns and mechanisms of human and mouse isoforms were discovered^8^. This underscores the importance of investigating the subcellular distribution of UBE3A in human specific models. While important previous studies examined the expression patterns of UBE3A in human cortical samples and hiPSC-derived neurons using the Sigma-Aldrich UBE3A antibody (SAB1404508)^11,13^, the specificity of this commonly used antibody has not been evaluated in human knockout models until recently^9^. Here we expanded upon this analysis and evaluated the specificities of the 7 antibodies in IF using H9_WT_ and H9_*UBE3A* m−/p−_ hESC lines (Fig. 2). Moreover, we also investigated the staining patterns in five additional hPSC lines including three AS models (Figure S2). Since the imprinting of the paternal allele is a characteristic specific to mature neurons not present in these cultures, we expected all undifferentiated stem cell lines including the AS models to express UBE3A.

Our results showed that commercial antibodies have different staining patterns in H9_WT_ hESCs and vary significantly in their background signal in H9_*UBE3A* m−/p−_ hESCs (Fig. 2). While Bethyl Laboratories antibodies showed a diffused staining pattern across nuclei and cytosol, the immunoreactivity of the Sigma-Aldrich antibodies was consistently enriched primarily in the cytosol across all hPSCs tested in this study (Figs. 2 and S2). Surprisingly, both Sigma-Aldrich UBE3A antibodies demonstrated significant and strong background signal in H9_*UBE3A* m−/p−_ hESCs (Fig. 2). In addition, the Bethyl Laboratories antibody targeting the E6-binding region (A300-352A) showed weaker labeling in H9_WT_ hESCs and higher nonspecific signal in H9_*UBE3A* m−/p−_ hESCs when used at the same concentration as the Bethyl Laboratories antibody targeting the region near the AZUL domain (A300-351A) (Figs. 1A,B, 2 and S2).

The Bio-Rad, Proteintech, and Novus Biologicals antibodies showed variable staining patterns. The Bio-Rad antibody was diffuse across nuclei and cytosol. The Proteintech antibody was primarily nuclear. The Novus Biologicals antibody had a stronger signal in the cytosol with a diffuse nuclear labeling (Figs. 2 and S2). All three of these antibodies also revealed strong staining signal in H9_*UBE3A* m−/p−_ hESCs (Fig. 2). Among the tested antibodies, Bethyl Laboratories UBE3A antibody (A300-351A) showed the lowest background in H9_*UBE3A* m−/p−_ hESCs compared to its signal in H9_WT_ hESCs (Fig. 2).

### Four commercial antibodies capture similar neurodevelopmental localization changes in differentiated hCOs

**Figure 3:**
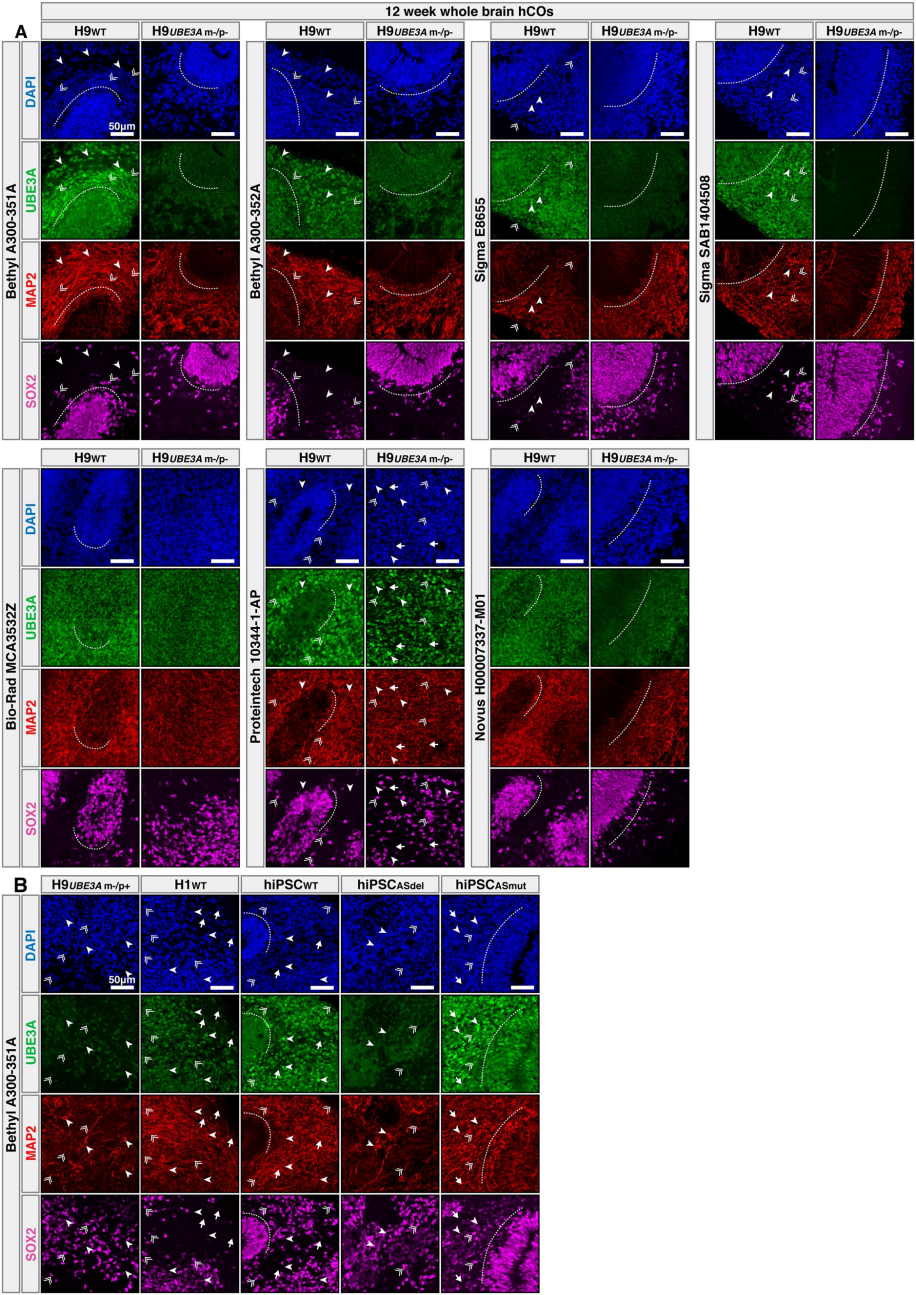
Four commercial antibodies capture similar neurodevelopmental localization patterns in differentiated hCOs. (**A**,**B**) Arrow heads: Neurons (MAP2+/SOX2−). Double arrows: Progenitor cells (SOX2+). Arrows: DAPI+ cells that are MAP2−/SOX2−/UBE3A−. (**A**) Immunofluorescence results from 12 week hCOs generated from H9 wild type (H9_WT_) and H9 double *UBE3A* knockout (H9_*UBE3 m−/p−*_) hESCs using seven different UBE3A antibodies. (**B**) Immunofluorescence results using antibody A300-351A on 12 week hCOs generated from H9 maternal *UBE3A* knockout hESCs (H9_*UBE3A* m−/p+_), wild type H1 hESCs (H1_WT_), hiPSCs derived from a neurotypical donor (hiPSC_WT_), hiPSCs derived from a patient with a large deletion in *UBE3A* region (hiPSC_ASdel_), and hiPSCs derived from a patient with a point mutation (F583S) in *UBE3A* (hiPSC_ASmut_).

In addition to hPSCs, we and others have previously used human stem cell-derived neurons^17,18^ and hCOs^15,19^ as promising experimental models for AS research. In particular, in a recent study our group showed that hCOs can recapitulate the important disease relevant molecular changes surrounding UBE3A including paternal imprinting and nuclear localization in neurons^15^. Here, we conducted IF experiments to evaluate not only antibody specificities, but also the ability of the selected antibodies to capture these salient molecular features of UBE3A using 12 week hCOs generated from seven different hPSC lines including three AS models (Figs. 3, S3 and S4).

In contrast to their different staining patterns observed during the pluripotent stage, both Bethyl Laboratories and both Sigma-Aldrich antibodies showed the expected nuclear staining pattern in neurons (MAP2+/SOX2−) of H9_WT_, hiPSC_WT_ and H1_WT_ hCOs (Figs. 3 and S3). Among these four antibodies, SAB1404508 showed the lowest background signal in H9_*UBE3A* m−/p−_ hCOs, A300-352A showed the highest background, while the nuclear localization pattern was the cleanest across all WT cell lines with A300-351A (Figs. 3 and S3A).

In addition, both Bethyl Laboratories antibodies showed reduced signal similar to their background levels in the neuronal regions (MAP2+/SOX2−) of both AS model hCOs (H9_*UBE3A* m−/p+_ and hiPSC_ASdel_) indicating imprinting and silencing of the paternal allele, as was previously observed^15^ (Figs. 3B and S3B). The signal from the Sigma-Aldrich antibodies in H9_*UBE3A* m−/p+_ and hiPSC_ASdel_ hCOs was stronger than their background levels, but it was reduced compared to WT hCOs, and the staining pattern appeared to be nonspecific particularly in neuronal regions (MAP2+/SOX2−) (Figure S3B). Collectively these results indicate that these four antibodies can capture the paternal imprinting of *UBE3A* in the neurons of hCOs.

Furthermore, all four antibodies also showed a striking nuclear localization pattern in the neurons of hiPSC_ASmut_ hCOs which was an anticipated result (Figs. 3B and S3B). Previously it was shown that UBE3A localization is regulated by the N-terminal extension and the AZUL domain^8,13^. The hiPSC_ASmut_ line was derived from a patient with a point mutation (F583S) on the N-terminal region of the N lobe of HECT domain, and previous reports indicate that this variant does not exhibit protein folding issues^20^. Furthermore, immunogens of the antibodies tested in this study do not directly align with the mutated region, with one exception being mouse monoclonal Sigma-Aldrich antibody (E8655) that was raised against full length human recombinant UBE3A (isoform1).

Bio-Rad, Proteintech and Novus Biologicals antibodies showed similar staining patterns and signal intensities across all hCO models including the H9_*UBE3A* m−/p−_ and AS models. Interestingly, while the staining patterns of the Bio-Rad and Novus Biologicals antibodies appeared to be diffuse and nonspecific, the Proteintech antibody exhibited strong nuclear labeling in neuronal regions of all hCO models (Figs. 3A and S3B). To ensure the elevated background signal observed with this antibody in H9_*UBE3A* m−/p−_ hCOs was not due to high antibody concentration, we conducted another IF assay using serial dilutions of this antibody in H9_WT_ and H9_*UBE3A* m−/p−_ hCOs. The results showed that even at low concentrations, the Proteintech UBE3A antibody showed similar labeling intensity between H9_WT_ and H9_*UBE3A* m−/p−_ hCOs (Figure S4A). Importantly, none of the secondary antibodies showed significant background labeling when used without the primary antibodies (Figure S4B).

### Four commercial antibodies show specific labeling of UBE3A in western blotting

Another commonly used method in the field to probe UBE3A expression is WB. While several important previous studies showed the specificity of Bethyl Laboratories and Sigma-Aldrich antibodies using adult AS mouse models^12,13,21–23^, the human specific activity of these antibodies in WB assays had not been tested previously with a double knockout cell line.

**Figure 4:**
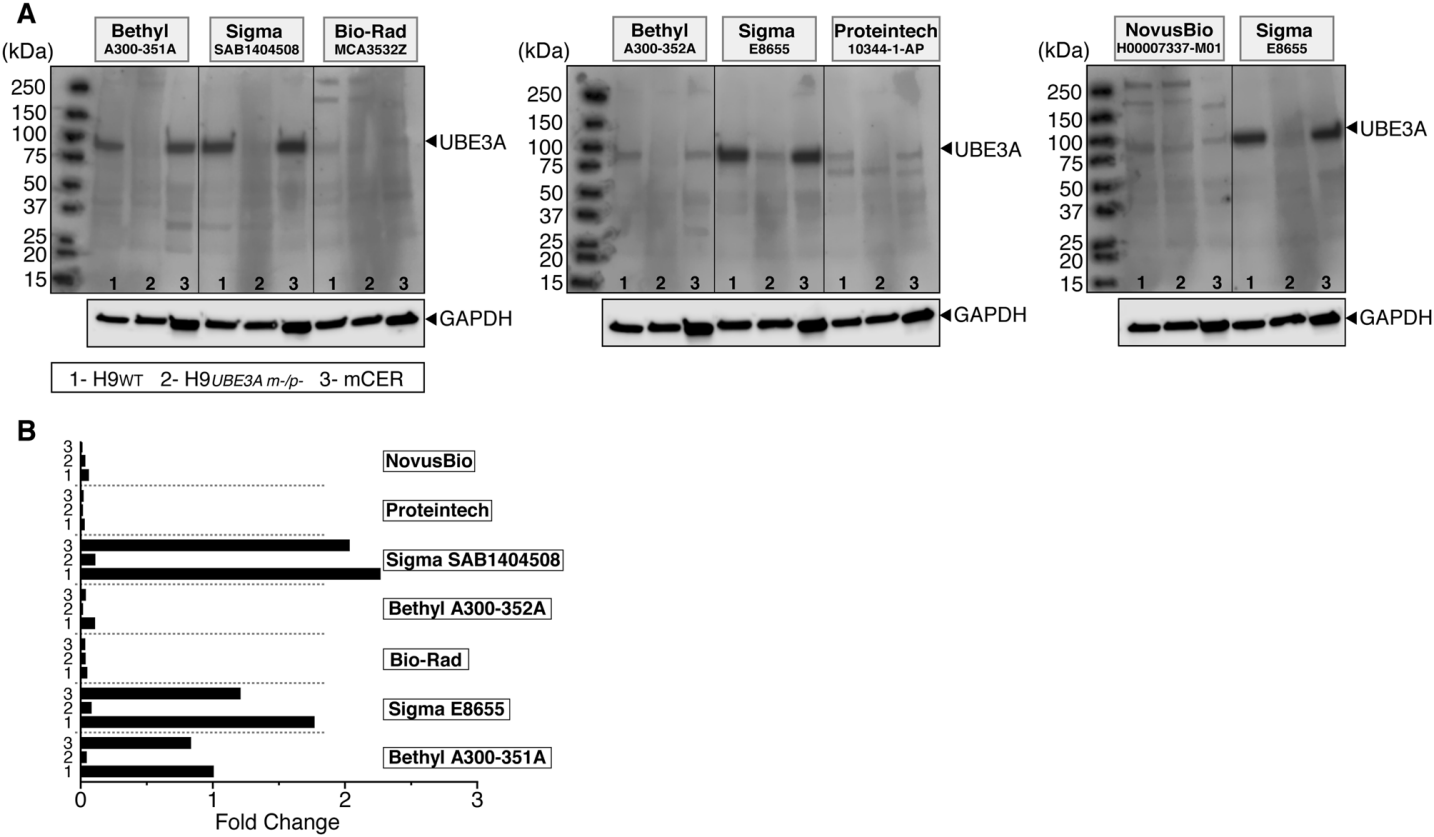
Four commercial antibodies show specific labeling of UBE3A in western blots. (**A**) Immunoblot analysis of UBE3A in whole cell lysates from H9 wild type (H9_WT_) hESCs, H9 double *UBE3A* knockout (H9_*UBE3A m−/p−*_) hESCs and wild-type mouse cerebellum (mCER) using seven different UBE3A antibodies. Full gels are depicted; vertical lines are drawn only for clarity. (**B**) Quantitative analysis of the UBE3A bands, normalized to GAPDH.

Here we used whole cell lysates from H9_WT_ and H9_*UBE3A* m−/p−_ hESCs to evaluate the specificity of the selected UBE3A antibodies in WB (Fig. 4). In addition to these cell lines, we also used WT adult mouse cerebellum (mCER) samples as controls (Fig. 4). Our results showed that both Bethyl Laboratories and both Sigma-Aldrich antibodies labeled specific bands in WT samples at ~ 100 kDa which is the predicted molecular weight for UBE3A (Uniprot: Q05086-1, 2, 3). While the Sigma-Aldrich antibody (E8655) showed a faint band in H9_*UBE3A* m−/p−_ hESCs lysate, when this condition was repeated in another blot, this band was much weaker and appeared as a smear in the membrane (Figs. 4A, S4C,D). In addition, low molecular weight, faint nonspecific bands were observable in the Bethyl Laboratories antibodies, particularly in the mCER samples (Fig. 4A). The Sigma-Aldrich antibody (SAB1404508) also showed weak, low molecular weight, nonspecific bands in H9_WT_ hESC samples. When used at the same concentration, Bethyl Laboratories antibody A300-352A showed significantly weaker staining compared to A300-351A which is in agreement with the IF results (Fig. 4).

The Bio-Rad and Novus Biologicals antibodies showed a similar band pattern to each other, with two higher molecular weight nonspecific bands in human cell lysates. The largest band was missing in mCER samples highlighting the species-specific differences in antibody reactivity (Fig. 4A). The Proteintech antibody also demonstrated nonspecific labeling around 75 kDa (Fig. 4A). The signal strength in the nonspecific bands observed with these three antibodies appeared to be comparable to the expected bands around 100 kDa, indicating that the Bio-Rad, Novus Biologicals and Proteintech antibodies have similar affinities towards UBE3A as they do to other off-target proteins.

## Discussion

During the last two decades, commercial antibodies have been widely used in scientific reports aimed to determine the abundance and subcellular distribution of UBE3A/Ube3a. While individual studies validated specific antibodies using AS and double knockout models in mice^12^ and human neurons^9^, the present work provides the first broad analysis dedicated to the evaluation of multiple commercial UBE3A antibodies in IF and WB assays using both mouse and human specific models that include multiple neural cell types.

Our analysis in mouse brain sections with the commonly used Sigma-Aldrich antibody (SAB1404508) showed that the IF protocol used in this study produced comparable results to previously published work^10,12^. Among the seven UBE3A antibodies tested, the Bethyl Laboratories antibodies (A300-351A and -352A) and another Sigma-Aldrich (E8655) antibody showed similar specific staining patterns in mouse brain tissue. However, while Bio-Rad and Novus Biologicals antibodies were raised against the same epitope sequence as the established Sigma-Aldrich antibody (SAB1404508), these antibodies revealed different patterns of reactivity with significant nonspecific signal in AS mice, potentially due to the differences in selected antibody clones (3E5 for Sigma-Aldrich SAB1404508, 2F6 for Bio-Rad and Novus Biologicals). While consistent with each other, Bio-Rad and Novus Biologicals antibodies also produced a distinct nonspecific band pattern in WB analysis with human hPSC lysates. These results indicate that these antibodies recognize unknown proteins of diverse molecular sizes other than UBE3A and highlight the importance of clone selection and validation in monoclonal antibody production.

Among the tested antibodies, the Proteintech antibody yielded the most unusual results. When tested in IF with adult WT mouse brain and WT hCO samples, it demonstrated the expected nuclear staining pattern in neuronal regions. However, it also showed the identical staining pattern and comparable signal strength in AS mouse sections and double knockout hCO sections. Consistent with this result, even though the WB analysis for the 100 kDa UBE3A target appeared as expected in WT lysate while being absent in the double UBE3A knockout samples, the Proteintech antibody also showed another band with similar signal strength to the UBE3A bands which appeared in both WT and double knockout cell lysates. These results indicate that the immunoreactivity observed with the Proteintech antibody is not solely related to the presence or absence of the UBE3A and that this antibody reacts with another distinct unknown protein. Although basic local alignment search tool (BLAST) analysis did not yield another potential target, the comparison of the epitope sequence used to raise this antibody with the protein coding UBE3A isoform sequences showed that it does not have 100% sequence homology with the most common UBE3A variant (hUBE3A iso1 and mUbe3a iso3) (Figs. 1A, B and S1A)^8,9^. This may imply a reduced affinity for the majority of the UBE3A protein expressed in a cell. Moreover, the polyclonal nature of this antibody may also contribute to its cross-reactivity with other targets. Due to its misleading IF staining pattern in WT models that looks like a specific signal, future studies that plan to utilize this antibody to determine UBE3A expression and localization should make sure the proper negative controls are employed.

Our IF analysis specifically in human hPSCs in their undifferentiated states revealed distinct staining patterns between the antibodies. These differences were stark, particularly between the polyclonal Bethyl Laboratories antibodies (diffuse cytoplasmic and nuclear staining) and the monoclonal Sigma-Aldrich antibodies (strongly cytoplasmic staining). In contrast, all four antibodies performed similarly in mouse brain sections and differentiated hCOs. Our analysis with the Sigma-Aldrich (SAB1404508) antibody also showed differences in staining pattern and background signal compared to a recently published study using the same double knockout hPSC line^9^. This discrepancy between the two studies could be due to differences in culture conditions (i.e. media reagents, feeder vs feeder-free conditions), where the morphology and gene expression state of pluripotent cells can vary substantially. This possible explanation is also supported by the results from hCOs where both Bethyl Laboratories and both Sigma-Aldrich antibodies demonstrated the expected nuclear staining pattern in the neurons of WT hCOs, captured indications of paternal imprinting in AS hCOs, and showed significantly reduced signal in hCOs generated from the double knockout hESC line.

This study highlights the importance of testing multiple UBE3A antibodies in different detection methods. The two Sigma-Aldrich antibodies have been the primary choice in the majority of the literature. Based on our results they are suitable for both IF and WB assays, albeit with a still unknown nonspecific signal in undifferentiated double UBE3A knockout hESCs. Both Bethyl Laboratories antibodies have previously been used in WB of mouse, hPSCs, and hPSC-derived neuronal lysates^9,24,25^; however, IF analysis with these antibodies was limited to a few studies in mouse models^21,26^. The present work indicates that the Bethyl A300-351A is a viable antibody option with performance at least on par with the Sigma-Aldrich antibodies and could be used for future IF and WB assays in both mouse and human model systems. Finally, it is not advised to use the Bio-Rad, Novus Biologicals, and Proteintech antibodies as they exhibit considerable non-specific staining in both IF and WB applications.

## Methods

### Cell culture and human cerebral organoid generation

Feeder-independent H9 and H1 hESCs (WA09 and WA01) were obtained from WiCell. Neurotypical hiPSCs were purchased from Systems Biosciences (Cat# SC102-A1). Angelman Syndrome (AS) hiPSCs with a class II deletion (AS_del_) were developed by the laboratories of Stormy J. Chamberlain and Marc Lalande at the University of Connecticut^27^ and obtained from Kerafast. H9_*UBE3A* m−/p−_ and H9_*UBE3A* m−/p+_ cells with a 66 kb deletion (chr15: 25338949–25405676) and AS point mutation hiPSC line (F583S) (AS_mut_) were provided by Dr. Stormy Chamberlain (UCONN). The H9_*UBE3Am−/p−*_ hESC cell line was generated and validated by Sirois and colleagues^9^. Our group also confirmed the deletion in these cells in an earlier study^15^. All cell lines were maintained in 6-well tissue culture dishes (Fisher Scientific) coated with 0.5 mg/cm^2^ Vitronectin (VTN-N) (Thermo Fisher) in E8 medium (Thermo Fisher) and passaged using standard protocols. Whole brain human cerebral organoids (hCOs) were generated and maintained using the same protocols as described^28^. Cells and hCOs were maintained in a humid incubator at 37 °C with 5% CO_2_.

### Animals

All methods to generate the original animal sections were carried out in accordance with the relevant guidelines and regulations of the University of North Carolina Institutional Animal Care and Use Committee. All experimental protocols using vertebrate animals to generate the original animal sections were approved by the University of North Carolina Institutional Animal Care and Use Committee. This study was carried out in compliance with the Animal Research: Reporting of in vivo Experiments (ARRIVE) guidelines. Wild-type (WT) (*Ube3a*_m+/p+_) and Angelman Syndrome (AS) (*Ube3a*_m−/p+_) mouse brain sections were residual samples from unrelated experiments and were provided by Dr. Ben Philpot (UNC). Mice carrying the *Ube3a* knock-out allele were originally generated by the laboratory of Dr. Arthur Beaudet^29^ and back-crossed ﻿ to a congenic C57BL/6J background. AS model mice were generated by mating WT male mice to female mice with paternal inheritance of the *Ube3a* knock-out allele (*Ube3a*_m+/p−_), which themselves are phenotypically normal^29^. Genotyping regarding the AS model mice has previously been described^10,12^. The sections used in this study are from a single perfused animal for each genotype (*Ube3a*_m+/p+_ and *Ube3a*_m−/p+_).

### Histology and immunofluorescence

Tissues and cells were fixed and immunostained as previously described^15^. Briefly, tissues and cells were fixed in 4% paraformaldehyde for 15 min at 4 °C followed by 3 × 10 min PBS washes. Tissues were allowed to sink in 30% sucrose overnight and then embedded in 10% gelatin/7.5% sucrose. Embedded tissues were frozen in an isopentane bath between − 50 and − 30 °C and stored at − 80 °C. Frozen blocks were cryosectioned to 30 µm. For immunohistochemistry, sections were blocked and permeabilized in 0.2% Triton X-100 and 5% normal donkey serum in PBS. Sections were incubated with primary antibodies in 0.2% Triton X-100, 5% normal donkey serum in PBS overnight at 4 °C in a humidity chamber. Sections were then incubated with secondary antibodies in 0.2% Triton X-100, 5% normal donkey serum in PBS for 2 h at room temperature, and nuclei were stained with DAPI (Invitrogen). Slides were mounted using ProLong Antifade Diamond (Thermo Fisher Scientific). Secondary antibodies used were donkey Alexa Fluor 488, 546 and 647 conjugates (Invitrogen, 1:250). Details on primary antibodies can be found below. Images were taken using a Nikon A1R confocal microscope (Nikon Instruments). High magnification images were captured using thin (1.5 μm) optical sections. All samples immunostained with the same antibody were processed at the same time, imaged using the same microscope settings, and adjusted identically. UBE3A antibody information including citations and antibody specifications provided by the vendors have been downloaded and stored on the Keung Lab GitHub page to provide access in the future if any of the antibodies used in this study are discontinued. The link to the publicly available GitHub repository is http://github.com/keung-lab/UBE3A_Antibodies.

Additional primary antibodies used in immunofluorescence assays:

**Table:** 

Antigen	Host	Supplier	Cat. No	RRID	Dilution
SOX2	Goat	R&D systems	AF2018	AB_355110	1:20
MAP2	Mouse	Millipore	M1406	AB_477171	1:250
MAP2	Rabbit	Millipore	AB5622	AB_91939	1:500

### Western blot

Whole cell lysates from H9_WT_, H9_*UBE3A* m−/p−_, and mouse cerebellum (mCER) derived from C57BL/6 mice were prepared as previously described^30^. Proteins separated in PAGE gel were transferred to PVDF membranes and treated with UBE3A antibodies (Figs. 1A and S1A). GAPDH was used as a loading control. The antigen–antibody complexes were visualized after incubation with the corresponding HRP-conjugated secondary antibody (anti-mouse-HRP and anti-rabbit-HRP) and ECL substrate (Bio-Rad, Clarity Western ECL Substrate). Signals from enhanced chemiluminescence were detected with a Licor Odyssey Fc imaging system using the Chemi channel after 10 min (UBE3A) and 5 min (GAPDH) exposures. The quantitative analysis was performed using Image Studio 5.2 Software (LI-COR Biosciences). While the membrane was cut vertically to allow for treatment with different UBE3A antibodies, they were treated together with ECL solution and imaged together using same exposure settings. Details on primary and secondary antibodies can be found below.

Primary and secondary antibodies used in immunoblotting assays:

**Table:** 

Antigen	Host	Supplier	Cat. No	RRID	Dilution
GAPDH	Mouse	Calbiochem	CB1001	AB_2107426	2 μg/ml
m-IgGκ BP-HRP	mouse IgGκ light chain	Santa Cruz Biotechnology	sc516102	AB_2687626	1:2500
r-IgG-HRP	Mouse anti-rabbit	Santa Cruz Biotechnology	sc2357	AB_628497	1:2500

## Supplementary Information

Supplementary Information.

## Data Availability

The datasets generated and/or analyzed during the current study are available from the corresponding author upon reasonable request.
