# Supplementary material for: Evaluation of UBE3A antibodies in mice and human cerebral organoids
Source: Sci Rep. 2021 Mar 18;11:6323. doi: 10.1038/s41598-021-85923-x (PMC7973473; doi:10.1038/s41598-021-85923-x)
Supplement: Supplementary file 1 — Supplementary Information. [file 41598_2021_85923_MOESM1_ESM.pdf]

Title: Evaluation of UBE3A antibodies in mice and human cerebral organoids

Authors: Dilara Sen[1], Zuzana Drobna[1,2], Albert J. Keung*[1, 3]

[1]Department of Chemical and Biomolecular Engineering, North Carolina State University, Raleigh, NC 27695-7905. [2]Department of Biological Sciences, North Carolina State University, Raleigh, NC 27695-7614. [3]Correspondance.

Corresponding author contact information

Albert Keung, PhD

CBE Department, North Carolina State University, Campus Box 7905, Raleigh, NC 27695-7905.

ajkeung@ncsu.edu

SUPPLEMENTAL FIGURES

A

Details of UBE3A antibodies						
					Dilutions & Concentrations	
#	UBE3A Antibody	Epitope Sequences	Clone	RRID	IF	WB
1	Bethyl Laboratories<br>Rabbit polyclonal<br>Cat# A300-351A	LYKINAKLCDPHPSKKGASSAYLENSKGAPNNSCSEIKMNKKGARIDFKDV	N/A	AB-185563	1:250	2 μg/mL
2	Bethyl Laboratories<br>Rabbit polyclonal<br>Cat# A300-352A	IPESSELTLQELLGEERRNKKGPRVDPLETELGVKTLDCRKPLIPFEEFI	N/A	AB-185564	1:250	2 μg/mL
3	Sigma Aldrich<br>Mouse monoclonal<br>Cat# E8655	MKRAAAKHLIERYYHQLTEGCGNEACTNEFCASCPTFLRMDNNAAAIKALELYKI<br>NAKLCDPHPSKKGASSAYLENSKGAPNNSCSEIKMNKKGARIDFKDVTYLTEEKV<br>YEILELCREREDYSPLIRVIGRVFSSAEALVQSFRKVKQHTKEELKSLQAKDEDKD<br>EDEKEKAACSAAAMEEDSEASSSRIGDSSQGDNNLQKLGPDDVSVDIDAIRRVY<br>TRLLSNEKIETAFLNALVYLSPNVECDLTYHNVYSRDPNYLNLFIIVMENRNLHSPE<br>YLEMALPLFCKAMSKLPLAAQGKLIRLWSKYNADQIRRMMETFQQLITYKVISNEF<br>NSRNLVNDDDAIVAASKCLKMVYYANVVGGEVDTNHNEEDDEEPIPESSELTLQ<br>ELLGEERRNKKGPRVDPLETELGVKTLDCRKPLIPFEEFINEPLNEVLEMDKDYTF<br>FKVETENKFSFMTCPFILNAVTKNLGLYYDNRIRMYSERRITVLYSLVQGQQLNPY<br>LRLKVRRDHIIDDALVRLEMIAMENPADLKKQLYVEFEGEQGVDEGGVSKEFFQL<br>VVEEIFNPDIGMFTYDESTKLFWFNPSSFETEGQFTLIGIVLGLAIYNNCILDVHFP<br>MVVYRKLMGKKGTFRDLGDSHPVLYQSLKDLLEYEGNVEDDMMITFQISQTDLF<br>GNPMMYDLKENGDKIPITNENRKEFVNLYSDYILNKSVEKQFKAFRRGFHMVTNE<br>SPLKYLFRPEEIELLICGSRNLDFQALEETTEYDGGYTRDSVLIREFWEIVHSFTDE<br>QKRLFLQFTTGTDRAPVGGLGKLKMIIAKNGPDTERLPTSHTCFNVLLLPEYSSKE<br>KLKERLLKAITYAKGFGML	330	AB-261956	1:100	2 μg/mL
4	Sigma Aldrich<br>Mouse monoclonal<br>Cat# SAB1404508	ETFQQLITYKVISNEFNSRNLVNDDDAIVAASKCLKMVYYANVVGGEVDTNHNEE<br>DDEEPIPESSELTLQELLGEERRNKKGPRVDPLETELGVKTLDCR	3E5	AB-10740376	1:500	2 μg/mL
5	Bio-Rad<br>Mouse monoclonal<br>Cat# MCA3532Z	ETFQQLITYKVISNEFNSRNLVNDDDAIVAASKCLKMVYYANVVGGEVDTNHNEE<br>DDEEPIPESSELTLQELLGEERRNKKGPRVDPLETELGVKTLDCR	2F6	AB-2211795	1:100	2 μg/mL
6	Proteintech<br>Rabbit polyclonal<br>Cat# 10344-1-AP	GEPQSDDIEASRMKRAAAKHLIERYYHQLTEGCGNEACTNEFCASCPTFLRMDN<br>NAAAIKALELYKINAKLCDPHPSKKGASSAYLENSKGAPNNSCSEIKMNKKGARID<br>FKDVTYLTEEKVYEILELCREREDYSPLIRVIGRVFSSAEALVQSFRKVKQHTKEE<br>LKSLQAKDEDKDEDEKEKAACSAAAMEEDSEASSSRIGDSS	N/A	AB-2211801	1:150	2 μg/mL
7	Novus Biologicals<br>Mouse monoclonal<br>Cat# H00007337-M01	ETFQQLITYKVISNEFNSRNLVNDDDAIVAASKCLKMVYYANVVGGEVDTNHNEE<br>DDEEPIPESSELTLQELLGEERRNKKGPRVDPLETELGVKTLDCR	2F6	AB-2241266	1:50	2 μg/mL

B

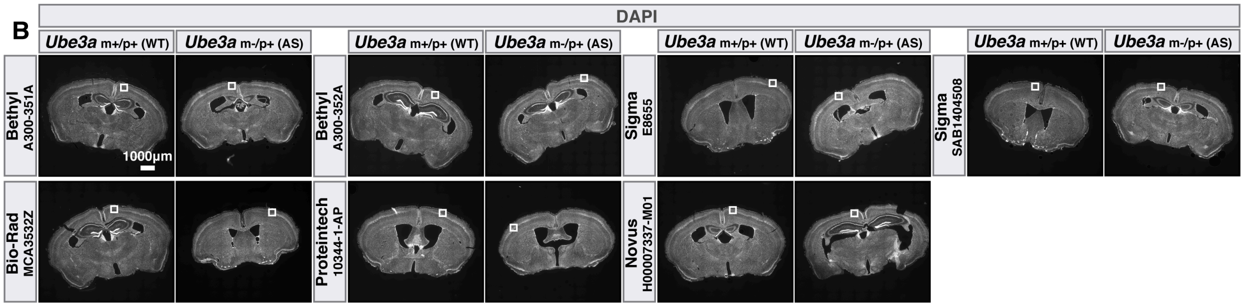

Figure S1. ***(Related to Figure 1)*** **Whole brain images from adult mouse brain sections used in this study.**

A) Epitope sequences and additional details for the seven different UBE3A antibodies used in this study. IF: Immunofluorescence, WB: Western blot.

B) A sample set of mouse brain sections used in this study. High magnification images of the boxed regions are depicted in Figure 1C.

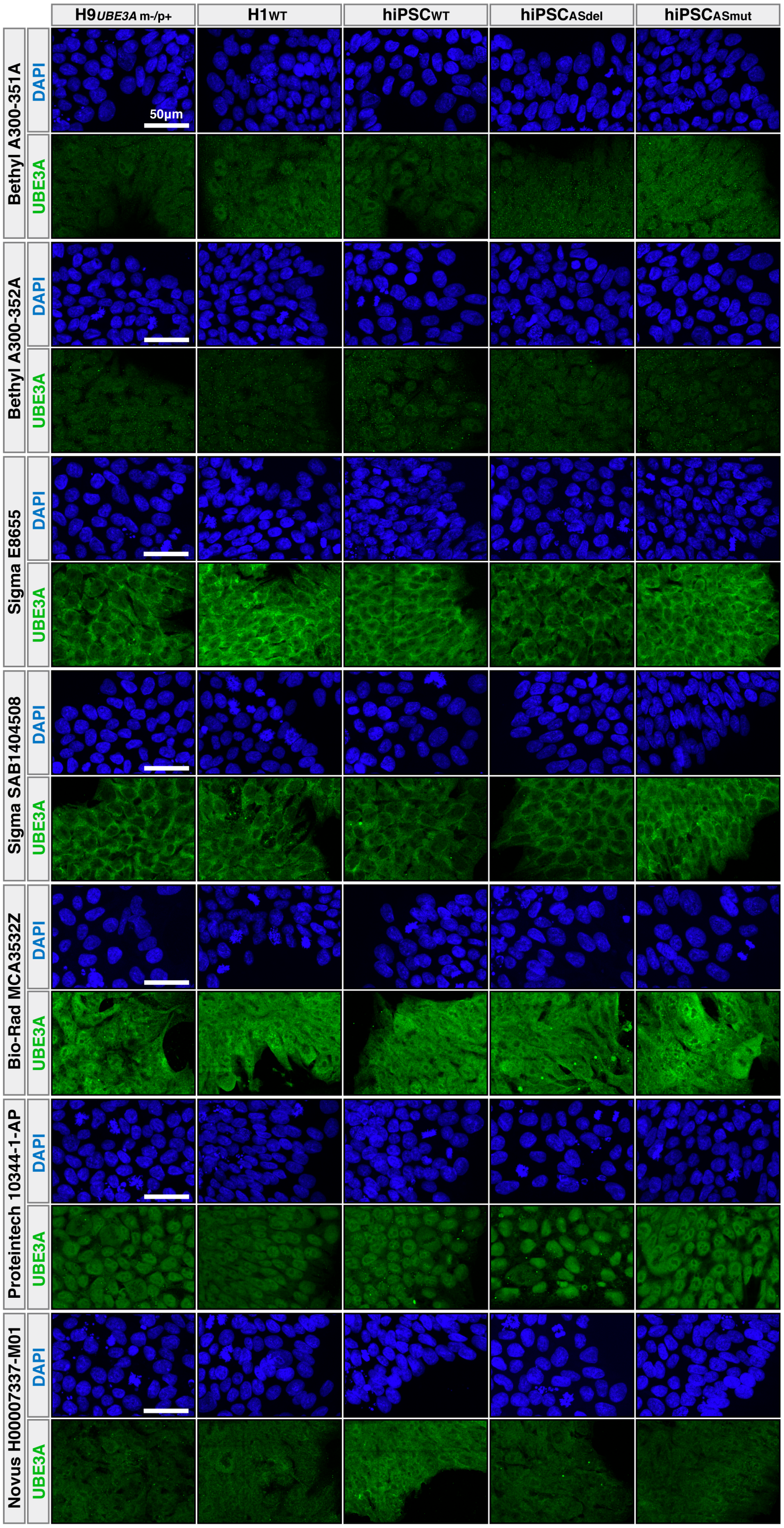

H9UBE3A m-/p+
H1WT
hiPSCWT
hiPSCASdel
hiPSCASmut
Bethyl A300-351A
Bethyl A300-352A
Sigma E8655
Sigma SAB1404508
Bio-Rad MCA3532Z
Proteintech 10344-1-AP
Novus H00007337-M01
DAPI
UBE3A
50µm

Figure S2. ***(Related to Figure 2)*** **Immunofluorescence analysis of additional hESC and iPSC lines stained with seven different UBE3A antibodies.**

H9 maternal *UBE3A* knockout hESCs ($H9_{\mathit{UBE3A}\ m-/p+}$), wild type H1 hESCs ($H1_{WT}$), hiPSCs derived from a neurotypical donor ($hiPSC_{WT}$), hiPSCs derived from a patient with a large deletion in *UBE3A* region ($hiPSC_{ASdel}$), hiPSCs derived from a patient with a point mutation (F583S) in *UBE3A* ($hiPSC_{ASmut}$).

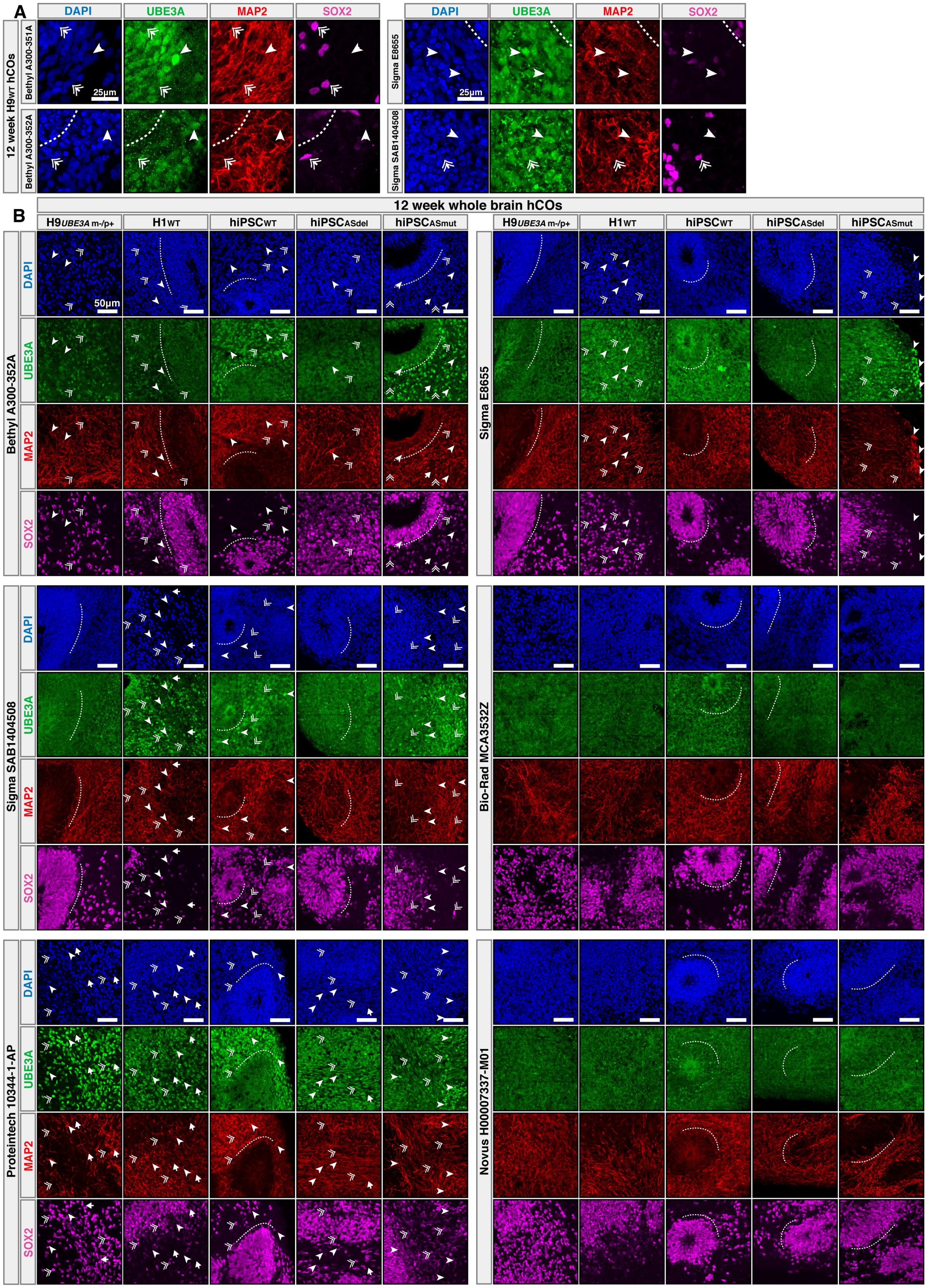

A
DAPI
UBE3A
MAP2
SOX2
12 week H9WT hCOs
Bethyl A300-351A
Bethyl A300-352A
Sigma E8655
Sigma SAB1404508
25µm
B
12 week whole brain hCOs
H9UBE3A m-/p+
H1WT
hiPSCWT
hiPSCASdel
hiPSCASmut
Bethyl A300-352A
Sigma E8655
Sigma SAB1404508
Bio-Rad MCA3532Z
Proteintech 10344-1-AP
Novus H00007337-M01
50µm

Figure S3. ***(Related to Figure 3)*** **Higher magnification images from Figure 3 and immunofluorescence analysis of additional hCOs derived from different hESC and hiPSC lines stained with six additional UBE3A antibodies.**

A) Digitally zoomed higher magnification images from Figure 3A showing the nuclear localization with both Bethyl Laboratories and Sigma Aldrich antibodies in 12 week $H9_{WT}$ hCOs.

B) H9 maternal *UBE3A* knockout hESCs ($H9_{\mathit{UBE3A}\ m-/p+}$), wild type H1 hESCs ($H1_{WT}$), hiPSCs derived from a neurotypical donor ($hiPSC_{WT}$), hiPSCs derived from a patient with a large deletion in *UBE3A* region ($hiPSC_{ASdel}$), hiPSCs derived from a patient with a point mutation (F583S) in *UBE3A* ($hiPSC_{ASmut}$). Arrow heads: Neurons (MAP2+/SOX2-). Double arrows: Progenitor cells (SOX2+). Arrows: DAPI+ cells that are MAP2-/SOX2-/UBE3A-.

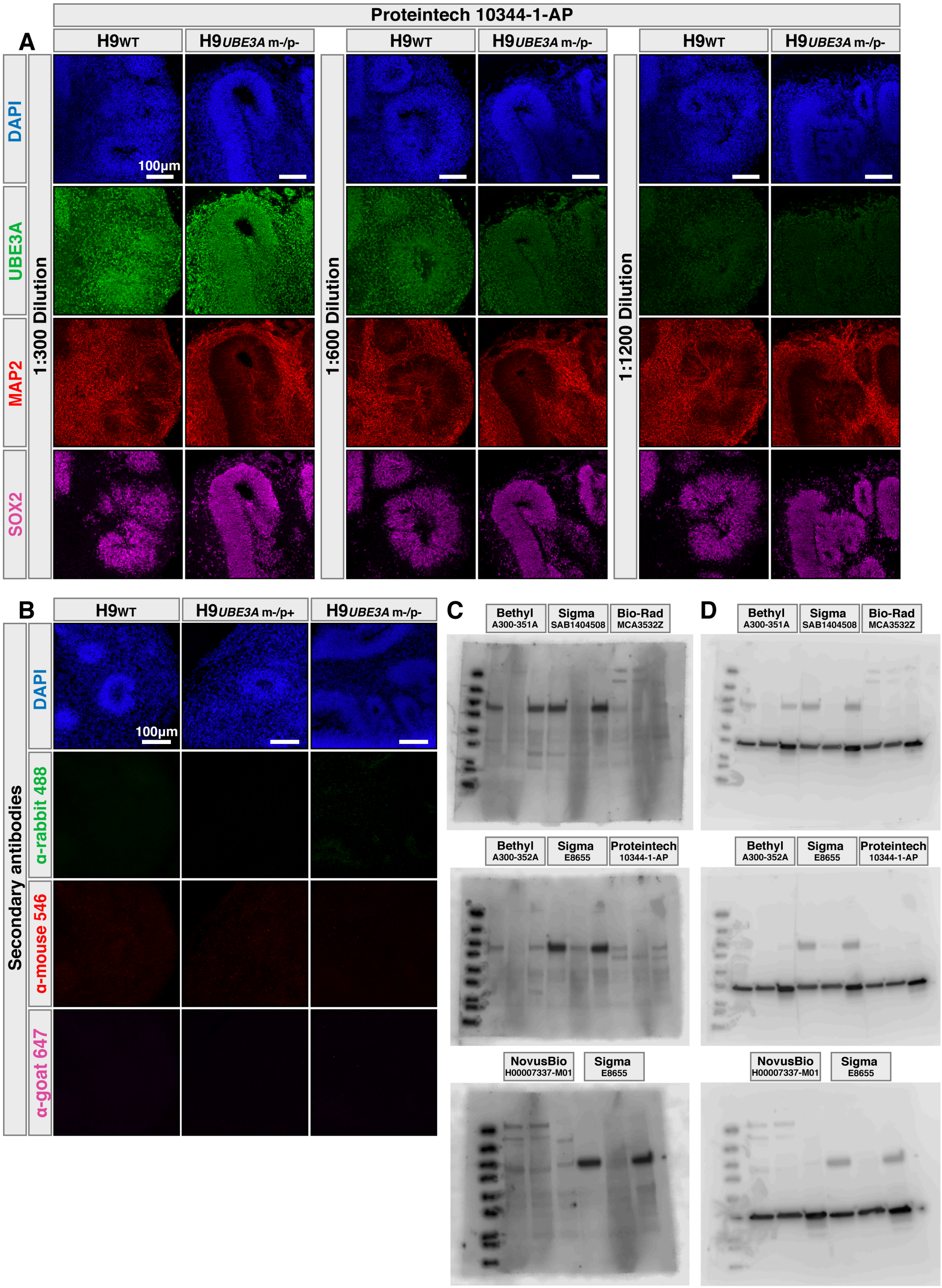

Proteintech 10344-1-AP
A
H9WT
H9UBE3A m-/p-
DAPI
UBE3A
MAP2
SOX2
1:300 Dilution
1:600 Dilution
1:1200 Dilution
100µm
B
H9WT
H9UBE3A m-/p+
H9UBE3A m-/p-
Secondary antibodies
DAPI
α-rabbit 488
α-mouse 546
α-goat 647
100µm
C
D
Bethyl A300-351A
Sigma SAB1404508
Bio-Rad MCA3532Z
Bethyl A300-352A
Sigma E8655
Proteintech 10344-1-AP
NovusBio H00007337-M01
Sigma E8655

Figure S4. ***(Related to Figure 3)*** **Titration of the Proteintech antibody, secondary antibody only immunofluorescence controls in hCOs, and unprocessed western blot images.**

A) 12 week hCOs generated from H9 wild type ($H9_{WT}$) and H9 double *UBE3A* knockout ($H9_{UBE3\ m-/p-}$) hESCs immunostained using different dilutions of Proteintech UBE3A antibody (10344-1-AP).
B) 12 week hCOs generated from H9 wild type ($H9_{WT}$), H9 maternal *UBE3A* knockout ($H9_{UBE3A\ m-/p+}$), and H9 double *UBE3A* knockout ($H9_{UBE3\ m-/p-}$) hESCs immunostained using only secondary antibodies.
C) Unprocessed western blot images for UBE3A staining showing the membrane edges.
D) Unprocessed western blot images for the loading control (GAPDH).
